# Regenerate to “Rejuvenate”: Insights From Adult Resident Stem Cells of Aged Flatworms and Mice

**DOI:** 10.1111/acel.70236

**Published:** 2025-09-14

**Authors:** Kevin A. Murach, Cory M. Dungan, Toby L. Chambers, Steve Horvath, Jayakrishnan Nandakumar, Vadim N. Gladyshev, Scott D. Pletcher, Xiaoting Dai, Longhua Guo

**Affiliations:** ^1^ Molecular Muscle Mass Regulation Laboratory, Department of Health, Human Performance, and Recreation University of Arkansas Fayetteville Arkansas USA; ^2^ Department of Health, Human Performance, and Recreation Baylor University Waco Texas USA; ^3^ Department of Human Genetics University of California los Angeles California Los Angeles USA; ^4^ Altos Labs San Diego California USA; ^5^ Department of Molecular, Cellular, and Developmental Biology University of Michigan Ann Arbor Michigan USA; ^6^ Division of Genetics, Department of Medicine Brigham and Women's Hospital, Harvard Medical School Boston Massachusetts USA; ^7^ Broad Institute Cambridge Massachusetts USA; ^8^ Department of Molecular & Integrative Physiology, Department of Cell & Developmental Biology University of Michigan Ann Arbor Michigan USA; ^9^ Institute of Gerontology, Geriatrics Center University of Michigan Ann Arbor Michigan USA

## Abstract

Adult resident stem cells are capable of regenerating tissues that manifest signs of “rejuvenation” in flatworms and mice of older ages. These findings suggest potentially conserved regulatory mechanisms of adult resident stem cells from worms to mammals. Regenerative capacities are more limited in specific tissues and stem cell types of larger mammals. Understanding and harnessing the rejuvenating properties of resident adult stem cells in flatworms and mice could have broad therapeutic implications for improving stem cell function and tissue plasticity across organ systems of humans in advanced age.

## Graphical abstract

Resident stem cells from aged planaria and murine skeletal muscle possess some inherent abilities to mitigate signs of aging after tissue regeneration.

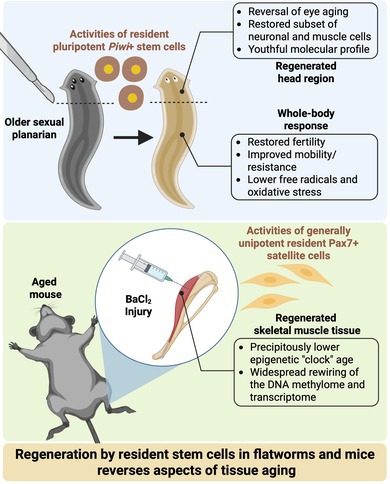

## 1 Regenerative Capacities of Resident Adult Stem Cells in Older Age

Aging is associated with numerous hallmarks that relate to structural and functional cell and tissue decline, and eventual mortality (López‐Otín et al. 2013, 2023). Stem cell exhaustion is one such hallmark that leads to diminished repair capacity after injury in regenerative tissues such as skeletal muscle (Brunet et al. 2023; Sousa‐Victor et al. 2022). Observations of a decline in adult (somatic) stem cell number and regenerative efficiency with aging has led to speculation on how replenishing stem cell pools may promote tissue “rejuvenation” through turnover of aged cells (Oh et al. 2014)—specifically in response to stress. We define rejuvenation as the reversal of age‐associated phenotypes or processes at the tissue or cellular level to that resembling a youthful state, or a significant alteration to molecular profiles that can result in youthful biological function and lower biological age. The transplantation of young stem cells combined with pharmacologic or biologic priming of the implantation niche is one such rejuvenation strategy. Unfortunately, this approach may not be scalable to all tissues and patients due to many barriers in the process: the generation of sufficient numbers of cells for transplantation, the promotion of stem cell survival and engraftment, potential immunological responses, and the viability of long‐term engraftment (Oh et al. 2014). Although adult mammalian stem cell attrition is commonly observed as a consequence of primary aging and/or secondary to declining activity levels, the remaining resident stem cells can still perform tissue adaptive functions after injury (Collins et al. 2007; Karlsen et al. 2020; Shavlakadze et al. 2010). Some adult stem cells may even be refractory to aging (Novak et al. 2021), and resident stem cells can still contribute to adaptation in geriatric animals (Thomas et al. 2025). Adult stem cell function is nevertheless hampered by the age‐associated inflammatory environment and dysregulation of immune cells (Blanc et al. 2025; Hoang et al. 2025; Shavlakadze et al. 2010). Reversion to a youthful inflammatory milieu in aged animals, by heterochronic parabiosis in rodents for example, can improve regenerative potential (Conboy et al. 2005); these findings point to some degree of inherent rejuvenating capacity within aged stem cells. The molecular revitalization of aged stem cells in rodents after heterochronic parabiosis is conserved across various tissue and stem cell types (Ma et al. 2022; Zhang et al. 2023). A provocative question therefore arises: do adult resident stem cells from aged organisms possess the capacity to regenerate a tissue that subsequently features signs of rejuvenation?

## 2 The Rejuvenating Potential of Planarian Stem Cells at Older Ages

**FIGURE 1:**
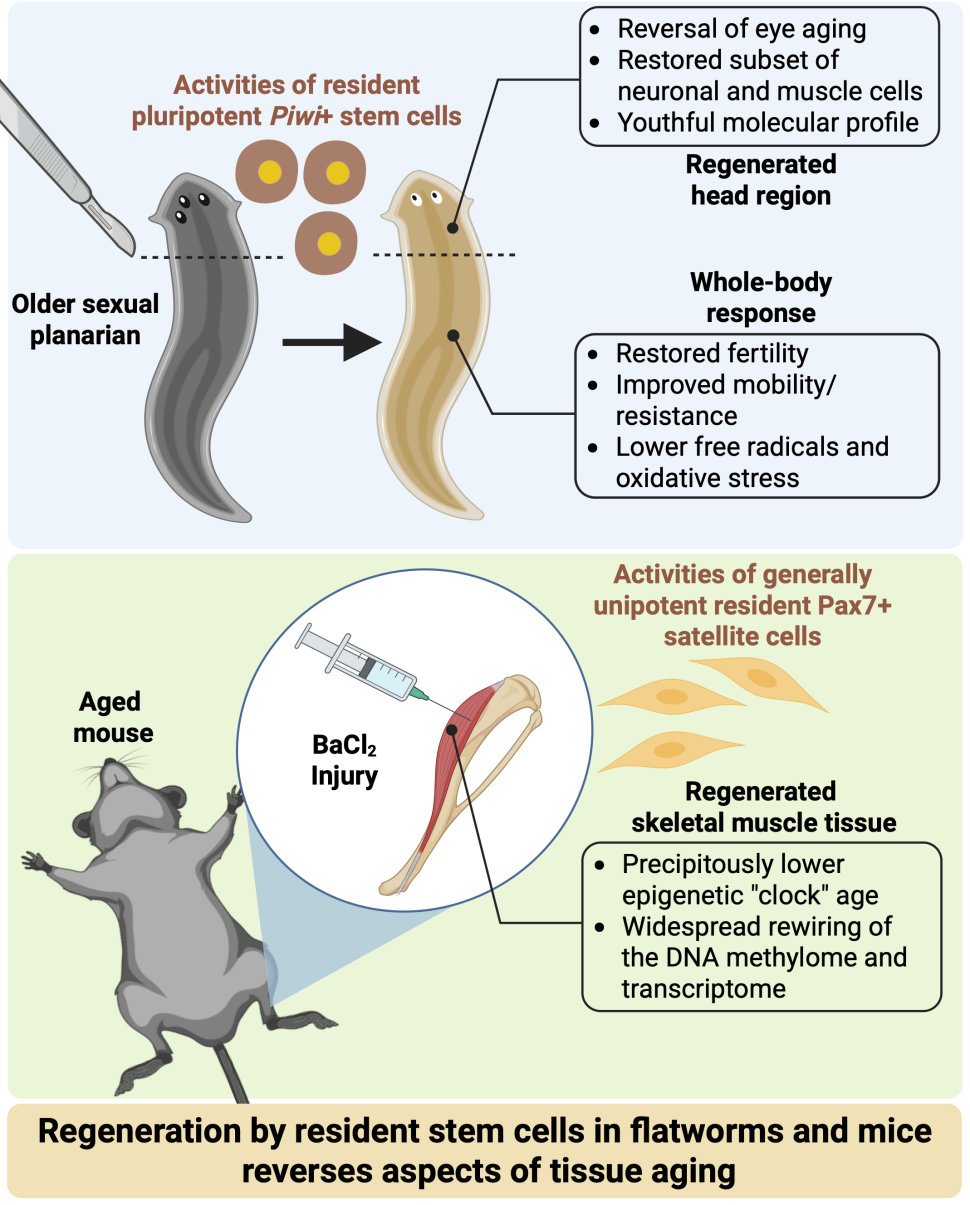
Summary figure illustrating the main findings related to signs of rejuvenation after regeneration in planarians (Dai et al. 2025) and mouse skeletal muscle (Chambers et al. 2025).

Planarians are considered “immortal” due to their extremely long lifespan and remarkable regenerative capacity (Austad 2009; Petralia et al. 2014; Sahu et al. 2017). Dai et al. recently used *Schmidtea mediterranea* ( *S. mediterranea* ) flatworms to study the effects of regeneration on aging (Dai et al. 2025). They found strains that proliferate via sexual reproduction manifest signs of aging at the molecular, cellular, and physiological levels. After phenotyping *S. mediterranea* to characterize signs of aging, Dai and coworkers reported that amputation of the heads in aged organisms and regeneration of new heads reversed aging of the eyes. This striking observation suggested that resident stem cells possess the rejuvenating capacity to reverse a common age‐associated dysfunction. Other physiological signs of aging such as reproductive infertility, impaired mobility, and increased free radicals and oxidative stress were also reversed by regeneration in older organisms. In the head, regeneration in older planarians restored a subset of neuronal and muscle cells that were lost during aging. At the molecular level, a planarian aging gene expression signature was reversed after regeneration. Tissue‐specific effects included changes to genes implicated in proteostasis (lysosome, protein folding), transcription and translation, oxidative stress, chromatin remodeling, and mitochondrial regulation. Disruptions to these processes are hallmarks of aging (López‐Otín et al. 2013, 2023). The regenerative ability of planarians was attributed to a pluripotent stem cell population that was enriched for the genes encoding *Piwi* (Reddien et al. 2005) and *Tert* (Dai et al. 2025). Collectively, these findings provide thought‐provoking evidence that resident adult stem cells can rejuvenate aged tissue to a more youthful state, especially in the newly regenerated heads of sexual planarians (Figure 1).

## 3 Epigenetic Rejuvenation With Injury Recovery in Aged Mammalian Skeletal Muscle

Skeletal muscle is an accessible solid tissue in humans that demonstrates overt signs of aging—reduced mass and strength/power producing capacity—which negatively impact mobility, independence, and mortality.

Certain muscles can be studied at the cellular and molecular levels in humans with relative ease via needle biopsy. Acessibility to skeletal muscle makes it an attractive tissue for exploring the effects of aging. Skeletal muscle is unique in that the primary cell type by volume, the muscle fiber, is multinucleated and muscle fiber nuclei (myonuclei) are nondividing organelles. It is also unique in that skeletal muscle is regenerative in mammals due to a relatively rare but highly replicative and typically (in nonpathological scenarios) unipotent resident stem cell population: Pax7+ satellite cells (Brack and Rando 2012). These cells decline in number with aging, which does not exacerbate age‐related sarcopenia, but are indispensable for muscle fiber regeneration across the lifespan (Fry et al. 2015; Keefe et al. 2015). Satellite cells typically reside in a quiescent (dormant) state until activated by an external simulus such as injury. They serve to reconstitute damaged muscle fibers, add to the myonuclear and mitochondrial pools during growth, and serve various paracrine functions (Goh et al. 2025; Murach, Dungan, et al. 2021; Murach, Fry, et al. 2021; Murach et al. 2018). Some evidence suggests that satellite cells can fuse to muscle fibers to replace damaged or dysfunctional myonuclei under basal homeostatic conditions (Keefe et al. 2015; Pawlikowski et al. 2015). However, aging and other conditions have been associated with myonuclear loss in some skeletal muscles throughout the lifespan (Bruusgaard et al. 2006; Keefe et al. 2015; Kirby and Dupont‐Versteegden 2022; Serrano et al. 2025), and prolonged satellite cell depletion does not cause or accentuate myonuclear loss by late life (Fry et al. 2015; Keefe et al. 2015; Englund et al. 2020). The prevalence of basal myonuclear turnover and its impact on muscle aging is therefore still unclear. Epigenetic dysregulation is a hallmark of aging (López‐Otín et al. 2013, 2023), and aging can be assessed via changes to DNA methylation age (DNAmAGE) which is widely used as a proxy for biological age across species (Horvath 2013; Horvath and Raj 2018; Lu et al. 2023). Changes to DNAmAGE can also be quantified in response to various exposures and interventions. Chambers et al. recently asked whether resident satellite cell‐dependent muscle regeneration could alter predicted DNAmAGE in aged murine muscle tissue (Chambers et al. 2025). It is perhaps intuitive that regeneration of aged murine skeletal muscle would not affect DNAmAGE of the tissue since the satellite cells (as well as supporting cell types) responsible for regeneration are the same chronological age as the organism. In other words, aged satellite cells should reconstruct an aged muscle fiber in all aspects. Surprisingly, 35 days after chemical injury in aged mice (24 months old), DNAmAGE of muscle tissue (i.e., all cell types combined) was precipitously decreased (Figure 1). This age reduction—up to 68% depending on the DNAmAGE clock used—is among the largest reported in the literature apart from epigenetic reprogramming by Yamanaka factors. Comparing old to young mice (4 months old) after regeneration, the magnitude of differential gene expression relative to uninjured was comparable and featured appreciable overlap; however, DNA methylation status and gene expression changes after injury were more tightly coupled in young muscle. This latter finding deserves further consideration since DNAmAGE was not lower after regeneration in young animals, and may have been accelerated depending on the clock used. Some epigenomic‐transcriptomic alterations linked to stem cell performance—upregulation of *Axin2*, *Egr1*, *Fzd4*, *Meg3*, and *Spry1*—were also unique to young muscle following injury recovery (Chambers et al. 2025).

## 4 Practical Implications of Resident Stem Cells Regenerating a “Younger” Muscle in Aged Mice

Some evidence suggests that repeated injuries enhance somatic stem cell performance (Falick Michaeli et al. 2022; Morroni et al. 2023), which provides information on adaptive cellular resiliency to stress. Alternatively, injury then recovery may cause impaired muscle tissue plasticity in response to a subsequent stressor in young skeletal muscle (Bigard et al. 2001; Kawano et al. 2017) along with aberrant extracellular matrix accumulation (Sato et al. 2003). A “rejuvenated” molecular signature after regeneration in aged muscle tissue (all cell types combined) points to a differential pace of aging depending on cell type—specifically the stem cells—within a tissue (Buckley et al. 2023; Gorelov et al. 2024). Initial attempts at quantifying the epigenetic profile of normally quiescent muscle stem cells indeed suggest that they do undergo methylation aging, but remain appreciably younger than most other tissues and cell types in animals (Hernando‐Herraez et al. 2019; Trapp et al. 2021) as well as the overall muscle tissue (Gorelov et al. 2024). Emerging evidence also suggests that forced proliferation of mammalian stem cells advances DNAmAGE (Gorelov et al. 2024). Myonuclei are nondividing organelles that have modest capacity for DNA synthesis (Borowik et al. 2022, 2024) but typically comprise the majority of all nuclei in muscle (Bagley et al. 2023; von Walden et al. 2020); and yet, skeletal muscle tissue still undergoes epigenetic aging on the aggregate (Chambers et al. 2025; Gorelov et al. 2024; Jones III et al. 2023; Murach et al. 2022). Perhaps epigenetic aging is more closely related to how transcriptionally active a nucleus is (i.e., its transcriptional “history”) versus whether or not it has undergone division. DNAmAGE reduction in muscle as the result of exercise training (Jones III et al. 2023; Murach et al. 2022) may in part depend on the contributions of typically unipotent satellite cells. These cells can elicit unique epigenetic effects in muscle after hypertrophic mechanical overload or lifelong wheel running (Murach, Dungan, et al. 2021; Murach et al. 2025).

## 5 The Rejuvenating Potential of Resident Stem Cells Across Species

In contrast to mammals, the resident stem cells of mature planarians (i.e., neoblasts) are pluripotent and can give rise to all body tissues (Dai et al. 2025; Fincher et al. 2018; Plass et al. 2018; Zeng et al. 2018). The numbers and transcriptional states of these stem cells manifest minimal changes in 3 year old planarians compared to young planarians (Dai et al. 2025). These findings contrast what occurs in murine muscle stem cells throughout their ~2 year lifespan (Kimmel et al. 2020; Lazure et al. 2023; Walter et al. 2024), as well as what is observed in aged human satellite cells (Kedlian et al. 2024; Lai et al. 2024). Unlike mammalian muscle stem cells, the planarian stem cell compartment can thus be largely maintained at youthful states as aging progresses, and for longer than the typical lifespan of a mouse. Recent work suggests that manipulating retinoic acid production can confer regenerative abilities to murine ear pinna (Lin et al. 2025). The importance of retinoic acid synthesis for stem cell performance is supported by evidence from regenerating zebrafish fins (Wehner et al. 2014) and aged murine myogenic cells (Fraczek et al. 2025). The possible benefits of retinoic acid signaling in highly regenerative axolotls has been recognized for some time (Maden 1982; Duerr et al. 2025; Khan et al. 2025). Whether retinoic acid treatment can elicit a rejuvenation effect is unclear. In young adult axolotls, it was recently observed that regeneration led to reduced DNAmAGE of newly formed limbs (Haluza et al. 2024). Epigenetic rejuvenation from tail regeneration remains unclear. It is important to note that limb fibroblastic cells are differentiated, and regeneration engages a major dedifferentiation process in which these cells acquire embryonic transcriptional states (Gerber et al. 2018). Interestingly, muscle cells are regenerated by Pax7+ stem cells in both limb (Sandoval‐Guzman et al. 2014; Fei et al. 2017) and tail (Wang et al. 2025). Additionally, Pax7+ stem cells in the tail are multipotent and can give rise to nonmuscle lineages (Wang et al. 2025). Whether rejuvenation can specifically occur in muscle tissues of both limb and tail after regeneration remains an open question in the axolotl (Haluza et al. 2024), especially in old adults. As tail regeneration does not engage the observed dedifferentiation process from the limb (Gerber et al. 2018) and is mainly driven by Pax7+ muscle stem cells and Meox1+ asomitic stem cells (Masselink et al. 2024), a closer examination of tail tissues for signs of rejuvenation will be intriguing. Based on these collective cross‐species examples, a deeper knowledge of how planarian stem cells maintain their “molecular youth” and superior regenerative potential over an extremely long relative lifespan, and understanding whether adult stem cells in aged axolotls confer rejuvenation effects, hold promise for guiding therapies and interventions that may further improve the performance of mammalian adult stem cells and adaptation in older age.

## 6 A Path Forward for Tissue Rejuvenation by Resident Stem Cells

The work discussed above raises several important considerations for future investigations: do molecular or cellular “rejuvenating” effects after regeneration vary according to tissue type, cell type, biological sex, or chronological age? Do single and repeated bouts of injuries induce the same extent of rejuvenation after regeneration? Does the magnitude of rejuvenation or aging mitigation differ after different time periods following regeneration (e.g., early versus late recovery)? To understand the differential effects of regeneration on DNAmAGE in younger and older mice is interesting. It is also worth considering the consequences of what molecular and cellular information may be lost as a result of resident stem cell‐mediated tissue rejuvenation (e.g., adaptive epigenetic memory, acquired cell resiliencies, and/or trained immunity), or potentially what could be gained (e.g., mutations specifically from within stem cells). With the capacity to induce regeneration in otherwise nonregenerative tissues in mammals (Lin et al. 2025), the notion of regeneration‐driven rejuvenation (Dai et al. 2025; Chambers et al. 2025) shifts from mere discoveries in model animals to a probable therapeutic approach in the future. We are not suggesting that injury should be used as a strategy to promote youthfulness. We instead posit that molecular pathways and cellular activities underlying regeneration‐induced rejuvenation can be used as a guide to enhance tissue health throughout the lifespan or in older populations, perhaps as an adjuvant to exercise or other therapies. Furthermore, since Pax7+ cells contribute to axolotl muscle regeneration (Sandoval‐Guzman et al. 2014; Fei et al. 2017), a more granular understanding of how these and other conserved stem cell populations compare to mammalian stem cells throughout the lifespan could unlock new triggers for healing or tissue youthfulness in humans (Dwaraka and Voss 2021). Hence, understanding the mechanisms of regeneration‐induced rejuvenation in model systems will be of paramount importance toward the goal of combatting human aging. Future work should aim to deeply phenotype whole tissues and organisms after regeneration in older ages across highly regenerative species such as spiny mice, axolotls, fish and reptiles. Determining whether regeneration‐induced mitigation of molecular and cellular aging profiles is followed by improved tissue plasticity and stress resilience is particularly important for translational and therapeutic applications. Collectively, recent work suggests that the plasticity of adult resident stem cells can be robust and that these cells possess some inherent capacity for facilitating youthful qualities of adult tissues at older ages.

## Author Contributions

This work was drafted by K.A.M. and revised by L.G. and K.A.M. The final version of the manuscript was edited and approved by all authors.

## Conflicts of Interest

The Regents of the University of California are the sole owners of patents and patent applications directed at epigenetic biomarkers for which Steve Horvath is a named inventor; S.H. is a founder and paid consultant of the nonprofit Epigenetic Clock Development Foundation that licenses these patents. S.H. is a Principal Investigator at Altos Labs, Cambridge Institute of Science, a biomedical company that works on rejuvenation.

## Data Availability

Data sharing not applicable to this article as no datasets were generated or analyzed during the current study.
